# Case report: novel mutations of *NDUFS6* and *NHLRC2* genes potentially cause the quick postnatal death of a Chinese Hani minority neonate with mitochondrial complex I deficiency and FINCA syndrome

**DOI:** 10.1097/MD.0000000000029239

**Published:** 2022-07-08

**Authors:** Yangfang Li, Yu Zhang, Gengpan Jiang, Yan Wang, Canlin He, Xiaofen Zhao, Ling Liu, Li Li

**Affiliations:** a Department of Neonatology, Kunming Children’s Hospital, Kunming 650228, Yunnan, China; b Kunming Key Laboratory of Children Infection and Immunity, Yunnan Key Laboratory of Children’s Major Disease Research, Yunnan Institute of Pediatrics, Kunming Children’s Hospital, Kunming 650228, Yunnan, China.

**Keywords:** FINCA syndrome, mitochondrial complex I deficiency, *NDUFS6*, neonate, *NHLRC2*

## Abstract

**Introduction:**

Mitochondrial complex I deficiency (MCID) and abbFINCA syndrome are lethal congenital diseases and cases in the neonatal period are rarely reported. Here, we identified a Chinese Hani minority neonate with rare MCID and FINCA syndrome. This study was to analyze the clinical manifestations and pathogenic gene variations, and to investigate causes of quick postnatal death of patient and possible molecular pathogenic mechanisms.

**Patient concerns:**

A 17-day-old patient had reduced muscle tension, diminished primitive reflexes, significantly abnormal blood gas analysis, and progressively increased blood lactate and blood glucose. Imaging studies revealed pneumonia, pulmonary hypertension, and brain abnormalities.

**Diagnosis:**

Whole-exome sequencing revealed that the *NDUFS6* gene of the patient carried c. 344G > T (p.C115F) novel homozygous variation, and the *NHLRC2* gene carried c. 1749C > G (p.F583L) and c. 2129C > T (p.T710M) novel compound heterozygous variation.

**Interventions and outcomes:**

The patient was given endotracheal intubation, respiratory support, high-frequency ventilation, antishock therapy, as well as iNO and Alprostadil to reduce pulmonary hypertension and maintain homeostatic equilibrium. However, the patient was critically ill and died in 27 days.

**Conclusion:**

The patient has MCID due to a novel mutation in *NDUFS6* and FINCA syndrome due to novel mutations in *NHLRC2*, which is the main reason for the rapid onset and quick death of the patient.

## 1. Introduction

Mitochondrial complex I deficiency (MCID) is one of the most common mitochondrial diseases,^[1]^ accounting for approximately 30% of human respiratory chain disorders, inherited in an autosomal recessive manner. Mitochondrial complex I (MCI) has a complex structure and comprises at least 45 subunits, of which 38 are encoded by the nucleus and the other 7 are encoded by mitochondria.^[2,3]^ MCID can result from a functional mutation in the genes encoding any of the subunits. It is reported that 20% of MCID is caused by mutations in genes encoded by mitochondrial DNA, and 80% is caused by mutations in nuclear genes.^[4–6]^ Due to the diversity of pathogenic genes, the clinical manifestations of MCID vary greatly among individuals, including lactic acidosis in the neonatal period, subacute necrotizing encephalomyelopathy (Leigh syndrome MIM: 256000) in early childhood, mitochondrial encephalomyopathy and MELAS syndrome in childhood, neurodegenerative diseases in adulthood, as well as cardiomyopathy, nephropathy, hepatopathy, leukodystrophy with macrocephaly.^[7,8]^ So far, 17 pathogenic genes of MCID have been included in the OMIM database,^[9]^ and there are still many other genes that may lead to MCID. However, the lack of relevant cases and studies regarding phenotype-genotype correlations makes the pathogenic molecular mechanism of MCID not fully clarified. *NDUFS6* is one of the known pathogenic genes of MCID on chromosome 5 (Chr 5q15.33) and consists of 4 exons and 3 introns. It encodes NADH dehydrogenase (ubiquinone) iron-sulfur protein 6, which is the main accessory subunit of MCI and involved in the transport of electrons from NADH to the respiratory chain.^[10]^ MCID resulting from *NDUFS6* deficiency often has an early onset and critical symptoms, with the most important clinical manifestation being fatal infantile lactic acidosis. Most of the patients die in the neonatal period.^[6]^ Animal studies have confirmed that Ndufs6 knockout in mice can lead to MCI deficiency, excessive ROS generation and kidney injury.^[11]^

FINCA (fibrosis, neurodegeneration, cerebral angiomatosis) syndrome is a progressive brain-lung disease (OMIM 618278) caused by a variation in the *NHLRC2* gene, first reported in 2018.^[12]^ FINCA syndrome is inherited in an autosomal recessive manner, with neurological symptoms first manifested, followed by multiple organ involvement as the disease progresses, such as severe tissue fibrosis, neurodegenerative diseases and cerebral hemangioma.^[13]^ The neuropathological findings of the patients include brain atrophy, vacuolar neurodegenerative diseases, loss of myelin with glioma, cerebral hemangioma, and neuronal loss in the anterior horn of the spinal cord.^[12]^ At present, there are few studies worldwide on FINCA syndrome, and the reported cases were diagnosed when patients developed relevant symptoms after 2 months of age, and there is no relevant report on the early manifestations of FINCA syndrome that may occur in the neonatal period. The pathogenic gene *NHLRC2* is located on chromosome 10 (Chr 10q25.3) and consists of 11 exons and 10 introns. *NHLRC2* encoded NHL-repeat-containing protein 2 is a 79-kDa protein containing 726 amino acids comprising an N-terminal thioredoxin (Trx)-like domain, a 6-bladed β-propeller domain, and a C-terminal β-strand domain. Studies have shown that there is a highly conserved cleft between the Trx-like domain and the β-propeller domain, which may be a binding site of NHLRC2 substrate or its interacting molecules, but no molecules interacting with NHLRC2 have been found so far.^[14]^ *NHLRC2* is evolutionarily highly conserved and may play an important role during brain development. High expression of *NHLRC2* mRNA can be detected early in both human and mouse brain development,^[15]^ while abnormal changes in NHLRC2 proteins and *NHLRC2* mRNA levels can be detected in patients with such neurodegenerative diseases as Parkinson disease and Alzheimer disease.^[16,17]^ Animal studies have confirmed that a homozygous mutation (p.Val311Ala) in the β-propeller domain of NHLRC2 can lead to a range of neural tube-related developmental malformations.^[18]^ In addition, NHLRC2 may be widely involved in embryonic development. In mice studies, X-gal staining revealed that Nhlrc2 is widely expressed in multiple organs during embryonic development, and complete loss of Nhlrc2 can lead to early embryonic death.^[12]^ Other studies have also found that variations of *NHLRC2* affect the cytoskeleton and vesicular trafficking of human skin fibroblasts, while FINCA patient-derived immortalized skin fibroblasts have a significantly enhanced ability to differentiate into myofibroblasts.^[19]^ In macrophages, NHLRC2 can act as a regulator and affect actin dynamics through the RhoA-Rac1 signaling pathway, thereby regulating phagocytosis.^[20]^ In colon cancer cells, loss of NHLRC2 can cause excessive reactive oxygen species (ROS) generation, thereby inducing apoptosis.^[21]^ Although a growing number of studies have confirmed the importance of *NHLRC2*, its specific physiological function remains unclear.

Both MCID and FINCA syndrome are extremely fatal genetic diseases. In view of the absence of effective therapeutic regimens, only symptomatic and supportive treatment is available. No cases of MCID with FINCA syndrome have been reported to date, especially FINCA syndrome is particularly poorly supported by case data from the neonatal period. This study investigates a Chinese Hani minority neonate with rare MCID and FINCA syndrome. We also analyze his clinical and biochemical characteristics and imaging findings, investigate the causes of his quick postnatal death, and explore the possible pathogenic molecular mechanisms and the genetic characteristics of the diseases in the Hani population. This study expands the pathogenic mutation map of MCID and FINCA syndrome and provides more basis for disease prevention, diagnosis and treatment.

## 2. Methods

The study was approved by the Ethics Committee of Kunming Children’s Hospital. All experiments were performed in compliance with the Helsinki Declaration. Informed written consent was obtained from the parents of the patient for the collection of clinical information, blood samples, DNA, and for presentation of patient’s materials.

### 2.1. Subjects

The proband was a Hani minority male infant born by spontaneous delivery (G7P6, 39W+4) and was admitted to the hospital at 17 days of age with a poor mental response for 4 days with fever. Physical examination on admission revealed a temperature of 37.5°C, heart rate of 147 beats/min, respiratory rate of 42 breaths/min, SPO_2_ of 82%– 92% (no oxygen inhaled), length of 54 cm, weight of 3.7 kg, and head circumference of 36 cm. The patient had poor general condition and response, little spontaneous activity, irritability after stimulation, flat and soft anterior fontanel (2 × 2 cm), mild yellowing of the skin, slight cyanosis around the mouth, glassy eyes, rough breathing sounds, reduced muscle tension, normal sucking reflex and grasp reflex, and diminished Moro reflex. During hospitalization, the child gradually developed pale limbs, poor limb circulation, reduced muscle tension, diminished primitive reflex, and significantly abnormal blood gas analysis. Therefore, he was given endotracheal intubation, respiratory support, high-frequency ventilation, antishock therapy, as well as iNO and Alprostadil to reduce pulmonary hypertension and maintain homeostatic equilibrium. However, the condition was not significantly improved, as blood lactate and blood glucose progressively increased and metabolic acidosis was difficult to correct. The patient died clinically 10 days after his hospitalization due to ineffective treatment, with an age of 27 days at the time of death. The proband’s parents were in good physical condition, all of them were of Hani population and denied consanguineous marriage and history of genetic diseases and infectious diseases, and the proband had 3 sisters in good health.

### 2.2. Clinical examination

The patient underwent systematic physical examination, laboratory tests, and imaging studies after admission. Physical examination mainly focused on primitive reflexes and muscle tension. Laboratory tests focused on arterial blood gas analysis. Imaging studies included UCG, brain MRI, and chest CT.

### 2.3. Whole-exome sequencing

A total of 2 ml peripheral blood (EDTA anticoagulant) was drawn from the patient, DNA was extracted from blood using a blood extraction kit, and 0.75 μg of DNA sample was taken for sonication to obtain 200–300 bp DNA fragments. A DNA library was constructed using KAPA Library Preparation Kit, and DNA concentration and purity were detected using NanoDrop2000. The biotin-labeled probe library DNA was hybridized under certain conditions; streptavidin magnetic beads were used to covalently bind biotin-labeled probes, so as to grasp target genes; finally, the magnetic beads carrying the target genes were adsorbed with a magnetic rack, eluted and purified; then, the target genes were enriched, and subject to Qubit quantification. The DNA samples captured were taken for Illumina NovaSeq high-throughput sequencing. After sequencing data were assessed to be qualified by Illumina Sequence Control Software (SCS), data reading and bioinformatics analysis were performed.

The results were verified by Sanger sequencing. Specific primers were used to amplify DNA fragments at the mutation site. Primers were used to amplify exon 4 of *NDUFS6* (NM_004553.4) (Chr5:1815999-F-1 CTGAGGTGTGGGGAGTGAAT;chr5:1815999-*R*-1TTGTCAGCCTTGACAGCAAC), exon 10 of *NHLRC2* (NM_198514.3) (Chr10:115664620-F-1 GGGTTTTGTTGTTGTCGCTAA, chr10:115664620-*R*-1TTTGATCCTGATGGGAGGTC) and exon 11 of *NHLRC2* (NM_198514.3) (Chr10:115668243-F-1GCAATGCCTGGCACTTAGAG;chr10:115668243-*R*-1 CAATGGGCATCTTGGTATCC). Details are as follows: Denaturation at 95°C for 5 min; denaturation at 95°C for 30 seconds, annealing at 65°C for 30 seconds, extension at 72°C for 30 seconds, 25 cycles in total, 0.6°C decreased in each cycle; denaturation at 95°C for 30 seconds, annealing at 50°C for 30 seconds, extension at 72°C for 1 min, 20 cycles in total; extension at 72°C for 10 min. When PCR was finished, 3 μL of the products were subjected to 2.5% agarose gel electrophoresis. PCR products were sequenced using an ABI 3730XL DNA analyzer.

## 3. Results

### 3.1. The disease of the patient progressed rapidly and the condition was critical

**Figure 1.**
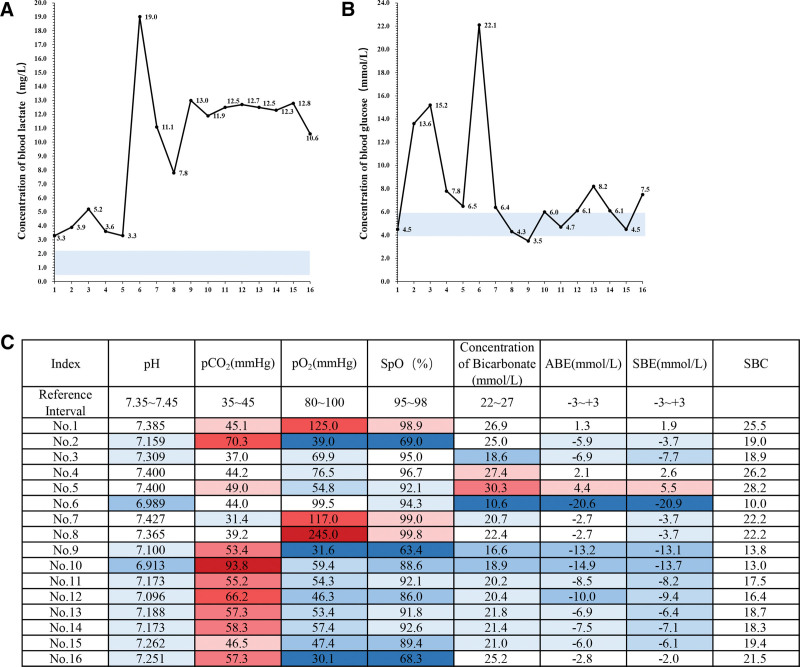
Blood lactate and glucose remained high in the patient. (A) The results of 16 times of blood lactate tests during the patient’s hospitalization. Blue block: normal range of blood lactate from 0.5 to 2.2 mg/L. (B) The results of 16 times of blood glucose tests during the patient’s hospitalization. Blue block: normal range of blood glucose from 3.9 to 5.9 mmol/L. (C) The patient had significantly abnormal blood gas analysis results. Red indicates a higher value than the normal range; blue indicates a lower value than the normal range. Darker color indicates more deviation from the normal range. ABE = actural base excess, pCO_2_ = partial pressure of carbon dioxide, pO_2_ = partial pressure of oxygen, SBC = standard bicarbonate radical, SBE = standard base excess, SpO = oxygen saturation.

**Figure 2.**
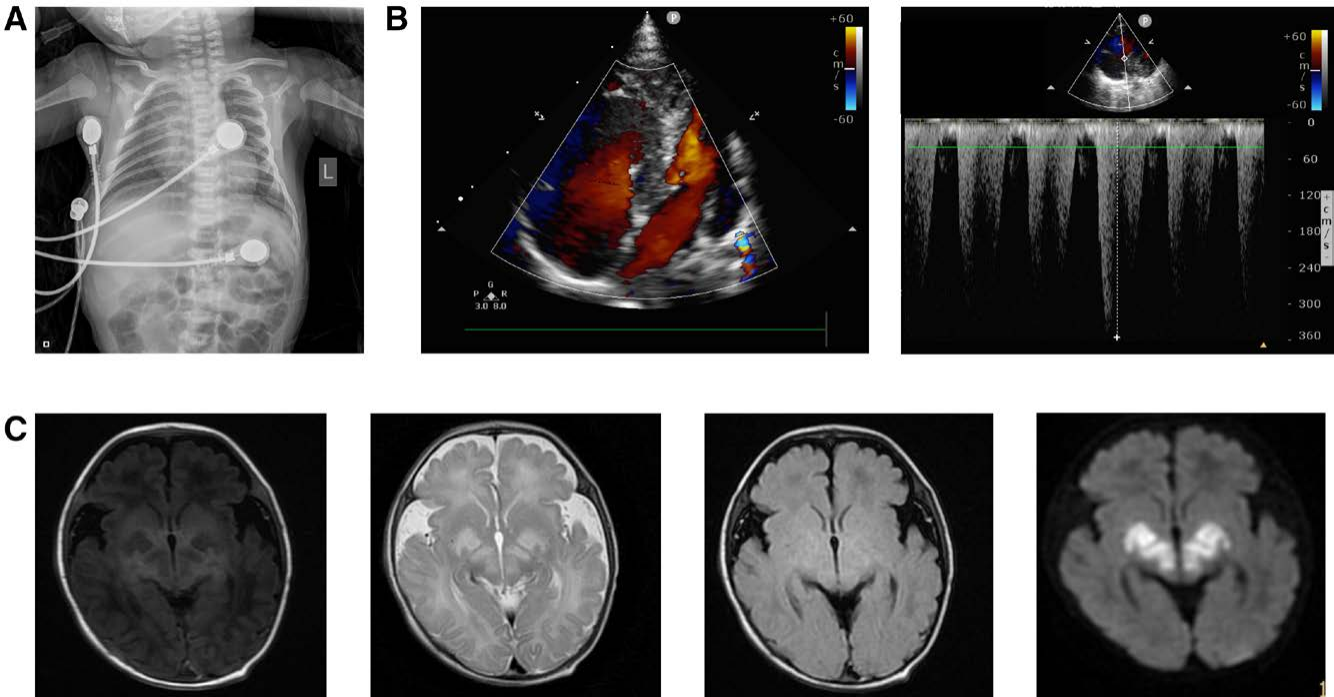
The patient suffered from neonatal pneumonia, right pulmonary hypertension, and possible leukodystrophy with CNS infection. (A) Chest CT findings. (B) UCG findings. (C) Brain MRI findings.

After admission, the patient underwent systematic physical examination, laboratory tests, and imaging studies. Physical examination showed poor response, little spontaneous activity, irritability after stimulation, slight cyanosis around the mouth, glassy eyes, diminished Moro reflex, shallow breathing, rough breathing sounds, progressive dyspnea, and reduced muscle tension. During hospitalization, he gradually developed pale limbs, further reduced muscle tension, and diminished primitive reflexes. Despite continuous treatment, laboratory arterial blood gas analysis showed significantly abnormal results, mainly manifested as high blood lactate concentration and blood glucose concentration. In normal subjects, the blood lactate concentration is 0.5–2.2 mg/L and the blood glucose concentration is 3.9–5.9 mmol/L. In this study, 16 times of blood gas analyses of the patient during hospitalization showed that the blood lactate concentrations were all higher than the normal range, up to 19.0 mg/L, about 9 times of normal subjects (Fig. 1A), and the blood glucose concentrations were also higher than the normal range for many times, up to 22.1 mmol/L, about 4 times of normal subjects (Fig. 1B). In addition, the partial pressure of carbon dioxide, oxygen partial pressure, bicarbonate concentration, actual base excess, standard base excess and standard bicarbonate radical fluctuated greatly, suggesting lactic acidosis, abnormal glucose metabolism and unstable vital signs (Fig. 1C). Bedside chest X-ray showed widened superior mediastinum considering thymic shadow, unclear structure of both hili, increased and blurred lung markings, and spot (plaque-like) high-density blurred shadows scattered in the middle and inner zones, suggesting that the patient suffered from neonatal pneumonia (Fig. 2A). Bedside UCG showed enlarged and bulged right atrium and right ventricle to the LV side, compressed outflow tract of left ventricle with pulmonary hypertension, and moderate tricuspid regurgitation, with an estimated PASP of 56 mm Hg (Fig. 2B). Brain MRI showed slightly higher water content in the white matter of the frontal, parietal, and temporal lobes, and slightly widened extracerebral space in the temporal region, suggesting that the child may have leukodystrophy and CNS infection (Fig. 2C).

### 3.2. The patient had mitochondrial complex I deficiency resulted from a novel homozygous mutation of *NDUFS6* gene c.344G> T (p.C115F)

**Figure 3.**
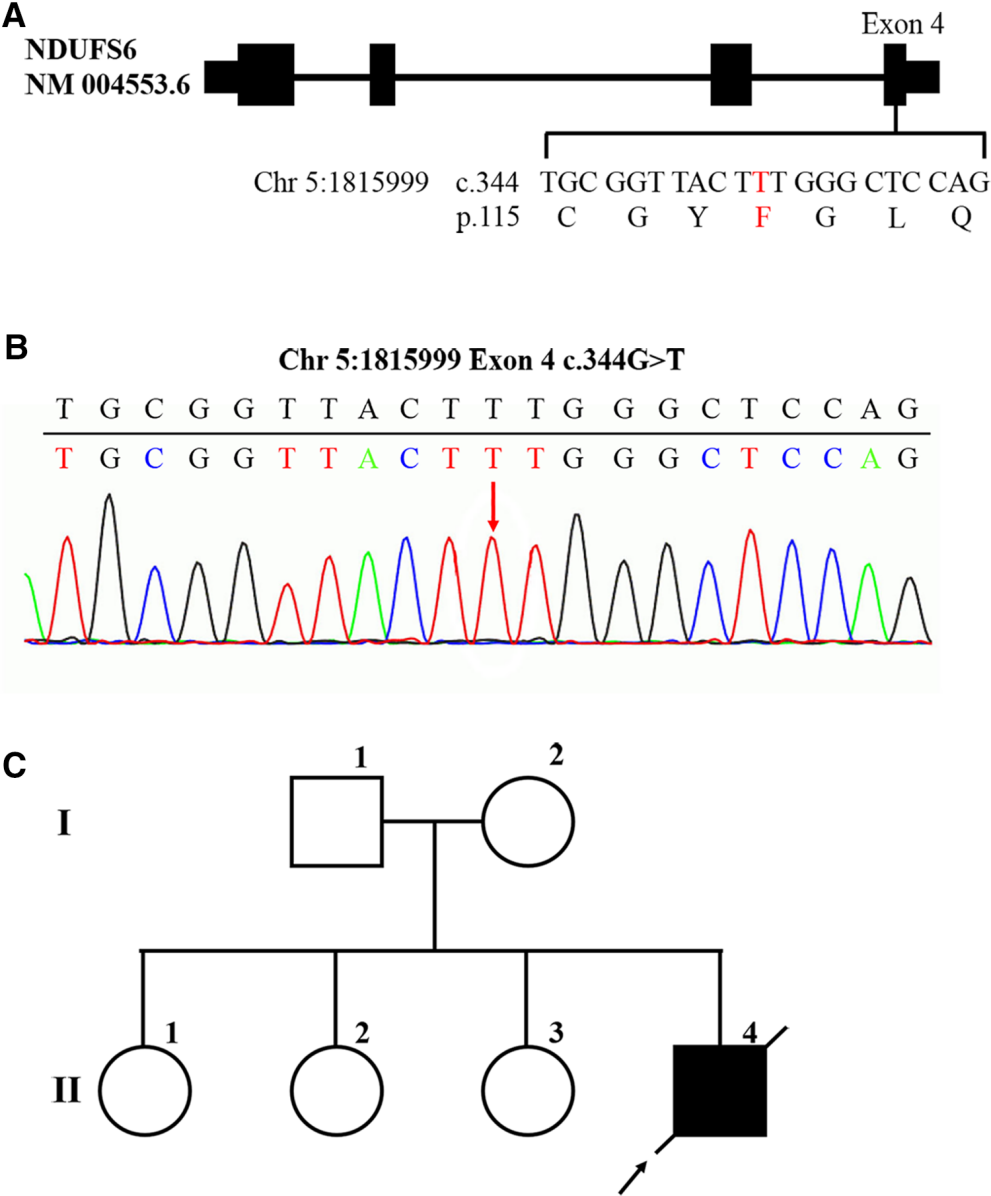
The patient had mitochondrial complex I deficiency resulted from a novel homozygous mutation of *NDUFS6* gene c.344G> T (p.C115F). (A) Diagram of *NDUFS6* gene and mutation location. The black rectangle indicates the exon region (drawn with reference to transcript NM004553.6). (B) Sanger first-generation sequencing verification results of *NDUFS6* gene mutations. The red arrow indicates the site of mutation. (C) Hereditary family tree of the patient.

During hospitalization, the symptoms of progressively increased blood lactate and blood glucose, and metabolic acidosis suggested that the patient was very likely to have inherited metabolic diseases. In order to detect whether the patient had abnormal genes, the peripheral blood DNA was extracted for Illumina NovaSeq high-throughput sequencing. The results showed a homozygous mutation in exon 4 of the *NDUFS6* gene, which substituted Guanine with Thymine at nucleotide 344 (c.344G > T), resulting in a substitution of Cysteine with Phenylalanine at amino acid 115 (p.C115F) (Fig. 3A). This mutation was verified by Sanger first-generation sequencing (Fig. 3B). Querying the HGMD database revealed that there have been studies reporting variations at the same amino acid position, but their nucleotide variant types were different from those in this study, so this is a novel mutation. Previous studies reported that c.344G > A homozygous mutation resulted in an amino acid change p.C115Y (Cysteine > Tyrosine) and triggered neonatal lactic acidosis, and c.343T > C homozygous mutation resulted in an amino acid change p. C115R (Cysteine > Arginine) and triggered Leigh syndrome. Both of the diseases were manifestations of MCID. Regarding the hereditary manner of MCID to be autosomal recessive and in combination with such symptoms as increased blood lactate and blood glucose and metabolic acidosis in the patient in this study, the patient could be identified as MCID resulted from a homozygous mutation in *NDUFS6* gene c.344G> T (p.C115F). The parents of the patient had no disease manifestations and were heterozygous carriers of this variant. The homozygous mutations carried by the patient were inherited from his parents, respectively, and his 3 3 sisters also had no disease phenotype and may or may not be heterozygous carriers of this variant (Fig. 3C). As the patient’s parents and 3 sisters refused to offer their DNA samples, the gene mutation of the patient’s family members could not be confirmed.

### 3.3. The patient had FINCA syndrome resulted from a novel compound heterozygous variation of *NHLRC2* gene c.1749C > G (p.F583L) and c.2129C > T (p.T710M)

**Figure 4.**
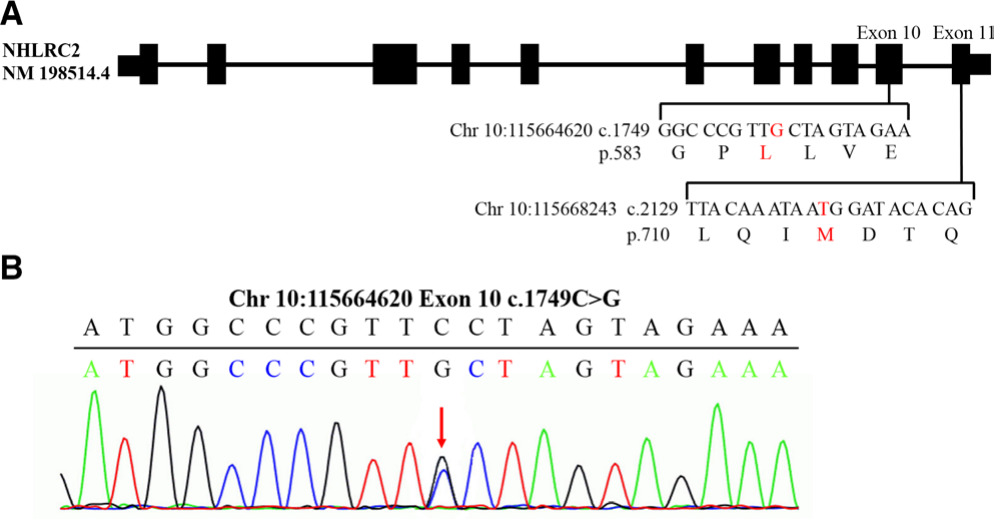
The patient may have FINCA syndrome resulted from a novel compound heterozygous mutation of *NHLRC2* gene c.1749C> G (p.F583L) and c.2129C> T (p.T710M). (A) Diagram of *NHLRC2* gene and mutation location. The black rectangle indicates the exon region (drawn with reference to transcript NM198514.4). (B) Sanger first-generation sequencing verification results of *NHLRC2* gene mutations. The red arrow indicates the site of mutation.

In addition to the abnormal *NDUFS6* gene, the whole-exome sequencing revealed another genetic variation. The patient had a compound heterozygous variation in exon 10 of *NHLRC2* gene, which substituted Cytosine with Guanine at nucleotide 1749 (c.1749C > G), resulting in a substitution of Phenylalanine with Leucine at amino acid 583 (p.F583L), and in exon 11, which substituted Cytosine with Thymine at nucleotide 2129 (c.2129C > T), resulting in a substitution of Threonine with Methionine at amino acid 710 (p.T710M) (Fig. 4A). The above mutations were verified by Sanger first-generation sequencing (Fig. 4B). Querying the HGMD database revealed that the mutations at the above 2 sites had not been reported in previous literature, so they were novel mutations. Brain MRI showed abnormal brain lesions, including slightly higher water content in the white matter of the frontal, parietal, and temporal lobes, and slightly widened extracerebral space in the temporal region, which may be early manifestations of FINCA syndrome in the neonatal period. So we speculated that the compound heterozygous mutation carried by the patient resulted in abnormal *NHLRC2* gene function and triggered FINCA syndrome. However, since the patient was critically ill and died in only 27 days, the subsequent neurological development could not be observed, so no more manifestations in the late course of FINCA syndrome could be observed.

## 4. Discussion

Mitochondrial complex I (MCI) has a complex structure and comprises more than 40 subunits. Assembly of such a complex structure requires the involvement of many assembly factors. It has been found that at least 15 proteins may be involved in MCI assembly. In addition to the defect of genes encoding MCI structure, the defect of genes encoding assembly factors can also lead to MCID.^[22]^ According to the reported cases, MCID is manifested as Leigh syndrome, myocardial encephalopathy, hepatic tubulopathy, leukodystrophy with macrocephaly, and severe neonatal lactic acidosis.^[8]^ Among them, Leigh syndrome is common in early childhood and resulted from mutations in NDUFS1, NDUFS3, NDUFS4, NDUFS7, NDUFS8, and NDUFV1 genes^[23]^; early-onset myocardial encephalopathy may result from mutations in NDUFS2 and NDUFV2 genes^[24]^; fatal infantile lactic acidosis may result from mutations in NDUFA11 gene.^[25]^ Even so, there are still many pathogenic genes of MCID that remain unknown and lack complete phenotype-genotype correlations, so MCID diagnosis by gene sequencing remains to be improved. Laboratory diagnosis of MCID is also very difficult, as biochemical parameters are often complex and overlapped and there are no specific features in general biochemical examinations. Studies have shown that the diagnosis can be made by the analysis of mitochondrial respiratory chain complex activity, but such significantly affected tissues as the brain, myocardium and endocrine glands must be collected by traumatic operations as ideal specimens, which is hard. It is rather unacceptable for neonates and their parents; so it is difficult to widely use in clinical practice. In this study, the patient was young, critically ill, and could not undergo tissue biopsy; so only gene sequencing could be selected for diagnosis. In addition, since the clinical manifestations of MCID have no obvious specificity compared with other mitochondrial diseases, the diagnosis of MCID should also be distinguished from other mitochondrial dysfunction diseases, especially multiple mitochondrial dysfunction syndrome (MMDS). MMDS is an autosomal recessive disease with clinical manifestations similar to MCID, such as leukodystrophy, decreased muscle tension, respiratory insufficiency, hyperglycemia, encephalopathy, neurological injuries, and dysplasia.^[26]^ However, MMDS has a different molecular mechanism of pathogenicity from MCID. MMDS is caused by nuclear gene mutations and can be divided into 6 types. Types 1–5 are caused by mutations in genes encoding the biosynthetic process of Fe-S clusters (ISC); type 6 is caused by mutations in genes encoding the catalytic subunits of mitochondrial processing proteases (MPP), which in turn impair mitochondrial function and present with a series of clinical manifestations.^[27,28]^ In summary, it is particularly important to constantly improve the pathogenic mutation spectrum of mitochondrial diseases and develop novel laboratory diagnosis and identification methods for mitochondrial diseases.

The patient in this study had MCID due to a novel homozygous mutation in *NDUFS6* and FINCA syndrome due to a novel compound heterozygous mutation in *NHLRC2*. The NDUFS6 protein is part of the hydrophilic peripheral arm of MCI and is located in the ISC of MCI.^[29]^ Studies have found that the activity of muscle tissue NADH (represents MCI) and cytochrome c oxidoreductase (represents mitochondrial complex III, MCIII) residues is severely reduced in MCID patients caused by NDUFS6 deficiency. It is speculated that NDUFS6 may be involved in the interaction between MCI and MCIII and play an important role in the late stage of MCI assembly process. The functional defect may affect the complete assembly of MCI or the peripheral arm stability of MCI.^[30]^ The pathogenic mutation carried by the patient was c.344G> T (p.C115F), where the original Cysteine is evolutionarily highly conserved. MCID due to variation at this site has also been reported in the past, and this mutation has a very low carrier rate in the normal population (<0.00001); so its variation may seriously affect protein function. The NHLRC2 protein contains 3 domains: N-terminal thioredoxin (Trx)-like domain, 6-bladed β-propeller domain, and C-terminal β-strand domain. Among them, the N-terminal Trx-like domain and 6-bladed β-propeller domain are more conserved among species. The CXXC motif site in the N-terminal Trx-like domain contains a CCINC motif, which is a typical feature of oxidoreductases and plays a role in thiol-disulfide exchanges. The 6-bladed β-propeller domain consists of 6 NHL repeats, and this structure has also been found in the NCL-1, HT2A, and LIN-41 genes, and is considered to be an important structure involved in protein interactions.^[31]^ Since the C-terminal β-strand domain is not conserved among species, few studies have focused on its function. The molecular pathogenic mechanism involved in FINCA syndrome induced by *NHLRC2* deficiency remains to be studied. Mice studies have founded that *NHLRC2* knockout can lead to increased hnRNP C2 expression in neurons and hippocampal pyramidal cells, accumulated RNA-binding proteins, and dysregulated RNA metabolism, ultimately causing neurodegenerative symptoms in FINCA syndrome.^[32]^ Two mutations c.1749C> G (p.F583L) and c.2129C> T (p.T710M) carried by the patient in this study were located in the C-terminal β-strand domain and may also be one of the reasons why neurodegenerative symptoms were not apparent in this patient. The carrier rate of the above 2 mutations in the normal population is extremely low (both <0.00005); so their effects on protein function are still worth studying. In this study, *NDUFS6* and *NHLRC2* were only subject to genetic testing, and clinical samples were not obtained for protein functional verification as the parents refused autopsy.

The simultaneous deficiency of *NDUFS6* and *NHLRC2* may be the main reason for the rapid onset and quick death of the patient. Some studies have found that NDUFS6 and NHLRC2 are both involved in the process of ROS generation in cells and mitochondria, and are associated with ROS-induced cellular senescence and apoptosis. MCI is known to be the main entrance of electrons into the respiratory chain and can regulate ROS, whose defects often lead to oxidative stress responses, allowing excessive increases of ROS and hindering normal intracellular ROS detoxification.^[33]^ Cells can produce ROS during normal metabolism or in a toxic state. Low levels of ROS can reversibly oxidize the thiol groups in proteins and modify the structure and function of proteins. However, excessive ROS will cause nonspecific damage to DNA, proteins, lipids and other macromolecules and induce cellular senescence and apoptosis, so it is very important to maintain the homeostasis of intracellular ROS.^[34]^ ROS are found to be significantly higher in fibroblast cells from MCID patients resulting from NDUFS6 deficiency.^[35]^ In mouse bone marrow mesenchymal stem cells, inhibition of NDUFS6 expression can lead to excessive ROS generation and excessive activation of p53/p21 signaling pathway, thereby accelerating cell senescence and apoptosis, while inhibition of ROS or supplementation of deficient NDUFS6 can slow down the senescence.^[36]^ NHLRC2 can also be involved in ROS-induced apoptosis, but its specific mechanism of action remains unclear. Studies in rectal cancer cells have found that excessive ROS can prompt the Thx-like domain of NHLRC2 to interact with caspase-8 zymogen, allowing NHLRC2 to be cleaved and degraded at Asp580, ultimately leading to apoptosis.^[21]^ Therefore, the simultaneous deficiency of the above 2 genes can lead to a complete dysregulation of ROS homeostasis in the patient, and whether there is a correlation between NDUFS6 and NHLRC2 protein function remains to be solved.

It is very rare that the patient suffers from 2 genetic diseases. Although his parents deny consanguineous marriage, they are both of the Chinese Hani minority population. The Hani minority originated from the nomads on the ancient Qinghai-Tibet Plateau and later migrated to the subtropical region of Yunnan Province. The Hani population is genetically and evolutionarily isolated due to its unique religious beliefs and living customs and tends to endogamy, resulting in a greatly increased proportion of genetic defects in its offspring. Future genome-wide association studies targeting ethnic minorities including the Hani minority can be performed to reveal the specificity of ethnic minority genomes and the molecular mechanisms underlying their high incidence of genetic diseases.

This study is the first report on neonatal MCID with FINCA syndrome, providing data support for the clinical manifestations in the neonatal period of both diseases. The mutations found in the study are novel which further expand the pathogenic mutation spectrum of MCID and FINCA syndrome and phenotype-genotype correlations, facilitate further investigation on the pathogenic molecular mechanisms of the 2 diseases, and provide more bases for the prevention, diagnosis, treatment and screening of the 2 diseases. However, as the patient is featured with rarity, rapid onset and quick death, pathogenesis studies about this patient are difficult to continue, and additional similar cases need to be collected for subsequent studies.

### Acknowledgment

We thank the patient and his parents for giving the permission to collect all clinical information, blood samples, DNA, and for presenting patient’s materials and test results in this study.

### Author contributions

Yangfang Li: Funding acquisition, Conceptualization. Yu Zhang: Investigation, Writing - Original Draft. Gengpan Jiang: Data Curation. Yan Wang: Data Curation. Canlin He: Resources. Xiaofen Zhao: Validation. Ling Liu: Methodology. Li Li: Writing - Review & Editing, Supervision, Funding acquisition.
